# Highly efficient and selective electrocatalytic hydrogen peroxide production on Co-O-C active centers on graphene oxide

**DOI:** 10.1038/s42004-022-00645-z

**Published:** 2022-03-28

**Authors:** Bin-Wei Zhang, Tao Zheng, Yun-Xiao Wang, Yi Du, Sheng-Qi Chu, Zhenhai Xia, Rose Amal, Shi-Xue Dou, Liming Dai

**Affiliations:** 1 Australian Carbon Materials Centre (A-CMC), School of Chemical Engineering, The University of New South Wales Sydney, Sydney, NSW 2052 Australia; 2 Department of Materials Science and Engineering, Department of Chemistry, University of North Texas, Denton, TX 76203 USA; 3 Institute for Superconducting and Electronic Materials, Australian Institute of Innovative Materials, University of Wollongong, Innovation Campus, Squires Way, North Wollongong, NSW 2500 Australia; 4 Beijing Synchrotron Radiation Facility, Institute of High Energy Physics, Chinese Academy of Sciences, Beijing, 100049 People’s Republic of China

**Keywords:** Electrocatalysis, Hydrogen fuel, Heterogeneous catalysis, Electrocatalysis

## Abstract

Electrochemical oxygen reduction provides an eco-friendly synthetic route to hydrogen peroxide (H_2_O_2_), a widely used green chemical. However, the kinetically sluggish and low-selectivity oxygen reduction reaction (ORR) is a key challenge to electrochemical production of H_2_O_2_ for practical applications. Herein, we demonstrate that single cobalt atoms anchored on oxygen functionalized graphene oxide form Co-O-C@GO active centres (abbreviated as Co_1_@GO for simplicity) that act as an efficient and durable electrocatalyst for H_2_O_2_ production. This Co_1_@GO electrocatalyst shows excellent electrochemical performance in O_2_-saturated 0.1 M KOH, exhibiting high reactivity with an onset potential of 0.91 V and H_2_O_2_ production of 1.0 mg cm^−2^ h^−1^ while affording high selectivity of 81.4% for H_2_O_2_. Our combined experimental observations and theoretical calculations indicate that the high reactivity and selectivity of Co_1_@GO for H_2_O_2_ electrogeneration arises from a synergistic effect between the O-bonded single Co atoms and adjacent oxygen functional groups (C-O bonds) of the GO present in the Co-O-C active centres.

## Abstract (web summary)

Hydrogen peroxide is an industrially highly demanded chemical, but its electrochemical synthesis still suffers from sluggish kinetics and imperfect selectivity. Here, the authors report a catalyst material comprising single cobalt atoms anchored on oxygen functionalized graphene oxide that produces 1.0 mg cm^−2^ h^−1^ of H_2_O_2_ with high selectivity and a low onset potential.

## Introduction

Hydrogen peroxide (H_2_O_2_) as one of the 100 most important chemicals in the world is widely used for various chemical and biological processes, including chemical and pharmaceutical production, environmental protection, and energy storage and conversion^1,2^. Currently, large-scale infrastructures and tedious multi-step processes are used for industrial production of H_2_O_2_ at a high temperature and pressure, thereby cost-intensive and rich with sewage by-product^3–6^. Thus, cost-effective and eco-friendly production of H_2_O_2_ is highly demanded^7–9^. In this context, the electrosynthesis of H_2_O_2_ through oxygen reduction reaction (ORR) via a two-electron pathway has recently attracted significant attention^10–14^. In particular, considerable effort has been made to enhance the activity and selectivity of electrocatalysts for the electrochemical production of H_2_O_2_^15,16^. However, the sluggish reactivity and low selectivity are still two obstacles for efficient electrosynthesis of H_2_O_2_ via the two-electron pathway of oxygen reduction.

Owing to their low coordination environment and high catalytic performance, carbon-based single-metal-atom catalysts (SACs) have recently attracted much attention for electrocatalysis^17–21^. In most SACs, however, single atoms are anchored on an N-doped carbon support to prevent migration of the metal atoms^22,23^, which usually lead to the four-electron pathway toward ORR^24,25^. Therefore, they are not ideal for electrosynthesis of H_2_O_2_, which requires a two-electron pathway toward ORR (i.e., 2e ORR)^26–30^. Compared to N-doped carbon materials, several oxygen functional groups, including COOH and C-O-C, in oxidized carbon materials have been proven to act as the defect-type less active sites for ORR via the two-electron pathway^31^. So, oxidized carbon materials can show good activity and selectivity for electrosynthesis of H_2_O_2_ by reducing O_2_ via a 2e ORR^31^. These studies prompt us to explore possible synergistic effects by anchoring SACs on oxidized carbon supports to boost the electrochemical production of H_2_O_2_ via 2e ORR.

Herein, we report an innovative approach to a class of SACs with single Co atoms anchored on graphene oxide of Co-O-C active centers (i.e., Co-O-C@GO, designed as Co_1_@GO for simplicity) with a high Co mass loading up to 1.8% with atomic dispersion. These single Co atoms form a Co-O bond with a bond length of 1.47 Å, resulting in a sufficiently high oxidation state for the Co sites for oxygen reduction via a two-electron pathway. As a result, the newly developed Co_1_@GO exhibited an excellent 2e ORR activity with an onset potential of 0.91 V and a high selectivity of 81.4% for H_2_O_2_ production. Of particular importance, the H_2_O_2_ yield of Co_1_@GO is 1.0 mg cm^−2^ h^−1^, which is a 19-fold enhancement compared with GO and about 2-fold improvement compared with Co nanoparticles/GO. Our combined experimental observations and density functional theory (DFT) calculations indicate that the high reactivity and selectivity of Co_1_@GO for electrogeneration of H_2_O_2_ is arising from the Co-O-C active center with synergistic effects between the O-bonded single Co atoms and adjacent oxygen functional groups (C-O bond) of the GO.

## Results and discussion

### The synthesis and structural characterization of Co_1_@GO

**Fig. 1:**
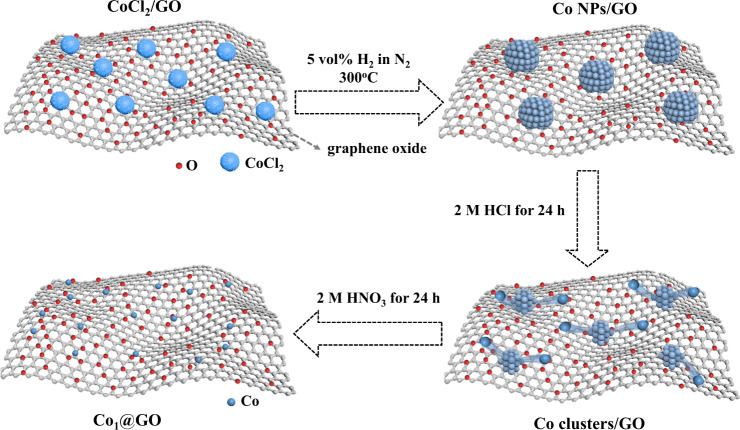
Schematic of synthesis of Co_1_@GO. Co nanoparticles were firstly prepared via reducing CoCl_2_ in 5 vol% H_2_ in N_2_ (Co NPs/GO). Co NPs/GO was leached to form Co_1_@GO.

**Fig. 2:**
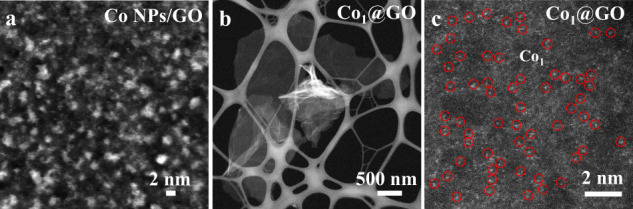
Electron microscopy images. HAADF-STEM images of **a** Co NPs/GO and **b**, **c** Co_1_@GO, with the red cycle areas of Co_1_.

Co_1_@GO was synthesized through acid leaching of Co nanoparticles (Co NPs/GO) (Figs. 1 and 2a and Supplementary Fig. 1), in which Co nanoparticles (~1.9 nm) were acid etched into Co clusters (Co clusters/GO) (Supplementary Fig. 2) and then etched into single Co atoms and stabilized by oxygen functional groups of the GO (Supplementary Fig. 3), leading to a stable Co_1_@GO electrocatalyst. It can be seen that the intermediate Co clusters/GO possesses Co clusters and single Co atom (Co_1_) (Supplementary Fig. 2c), though dominated by the Co clusters. The presence of Co_1_ suggested that part of Co clusters were dissolved by acid leaching to form single Co atoms, which were captured by the oxygen functional group of GO via Co-O bonding (Fig. 1). Further acid etching removed the Co clusters. High-angle annular dark field scanning transmission electron microscope images of Co_1_@GO are given in Fig. 2b, c and Supplementary Fig. 3, which show no Co NPs or Co clusters, but single Co atoms homogeneously interdispersed onto the GO support. Figure 2a, b reveals that Co clusters dissolved by acid leaching to form single Co atoms while Fig. 2c suggests that the resultant single Co atoms are stabilized on the GO support by Co-O bonding (vide infra). The energy-dispersive X-ray spectroscopy result in Supplementary Fig. S3c shows the presence of Co element in GO, further referring to the stability of single Co atoms anchored on the GO support. The X-ray diffraction (XRD) pattern of Co_1_@GO does not reveal additional diffraction peaks of Co apart from the typical broad peak centered around 24.9° from GO (Supplementary Fig. 4), indicating the successful transformation of the Co NPs to single Co atoms in Co_1_@GO (cf. Figs. 1 and 2 and Supplementary Figs. 1 and 2). The mass loading of Co in Co_1_@GO was determined by inductively coupled plasma–optical emission spectroscopic measurements to be about 1.8%. To investigate the GO structure of Co_1_@GO, we first performed the Raman spectroscopic measurements. As shown in Supplementary Fig. 5, the D band and G band of Co_1_@GO are at 1342.33 and 1587.49 cm^−1^, respectively, similar to those for GO, which indicates that acid leaching did not cause an obvious change of the GO structure in Co_1_@GO^32,33^. The Co 2p XPS spectrum of Co NPs/GO (Supplementary Fig. 6) could be separated into Co^2+^ (780.9 eV) to Co^3+^ (782.7 eV). The Co^2+^ (780.9 eV) state indicates that the surface of Co NPs has been oxidized into CoO. The Co^2+^ (781.1 eV) state of Co clusters/GO shows a positive shift of 0.2 eV compared with that of Co NPs/GO, which suggests that, after the HCl etching, the surface of Co clusters adsorbs hydroxyl (-OH). It could be confirmed that the C-O state of Co clusters/GO (533.5 eV) shows a down-shift of 0.2 eV compared with Co NPs/GO (C-O 1s, 533.7 eV), indicating that it has -OH on the surface of Co clusters and GO. The Co 2p XPS spectra of Co_1_@GO (Supplementary Fig. 6c) indicate that the Co atom site in Co_1_@GO is at Co^2+^ to Co^3+^, suggesting the presence of Co-O bond. The high-resolution O1s XPS spectrum of Co_1_@GO (Supplementary Fig. 6f) shows three peaks: C-O-C (533.6 eV), C-O-H (532.5 eV) and C=O (531.1 eV). The C1s XPS spectra of these three samples also demonstrate the presence of C-O-C, C-O-H and C=O (Supplementary Fig. 6g–i). In addition, the ratio of C-O-C:C-O-H:C=O in Co NPs/GO, Co clusters/GO, and Co_1_@GO are 0.8:0.6:1, 1.7:1.4:1, and 2.3:1.5:1, respectively. The high content of C-O-C in Co_1_@GO suggests the high selectivity for H_2_O_2_^31^. Interestingly, the C-O-C bind energy in O1s peaks of Co_1_@GO is the lowest among these three samples, which indicated that the high oxide state of single-atom Co may contribute electron to the C-O, leading to the formation of a Co-O-C electron conjugated complex. The as-measured oxygen contents of Co NPs/GO, Co clusters/GO, and Co_1_@GO are 12.3%, 14.5% and 15.3%, respectively. The presence of the Co-O-C electron conjugated complex and the high oxygen content in Co_1_@GO suggests the high performance of two-electron pathway for ORR.

**Fig. 3:**
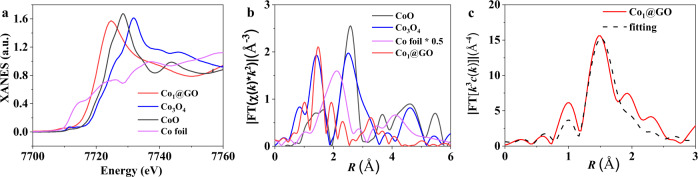
X-ray absorption results of Co_1_@GO. **a** XANES spectra and **b** Fourier transforms (FTs) of the Co K-edge of Co_1_@GO, Co foil, CoO and Co_3_O_4_. **c** Corresponding fitting of the EXAFS spectra of Co_1_@GO in R space.

The presence of single Co atom on GO was further confirmed by X-ray absorption spectroscopic (XAS) measurements. The X-ray absorption near-edge structure spectra in Fig. 3a shows that the absorption edge intensity of Co_1_@GO is higher than that of Co foil and close to those of Co_3_O_4_ and CoO. In addition, the absorption edge peak position of Co_1_@GO is positively shifted compared with those of Co_3_O_4_ and CoO. These results indicate that Co is atomically dispersed in the Co_1_@GO, and that the Co center is coordinated with O atoms by interacting with oxygen functional groups in the GO support to form the Co-O bond^34–36^. The Fourier-transformed k^3^-weighted extended X-ray absorption fine structure spectra of Co_1_@GO, Co_3_O_4_, CoO, and Co foil are reproduced in Fig. 3b, which clearly show no notable peak from 2.0 to 3.0 Å for the Co-Co bond in Co_1_@GO, but a prominent peak at 1.47 Å for the Co-O bond^37^. Clearly, therefore, the Co NPs have been dissolved and remained as single Co atoms to coordinate with O (Co-O-C electron conjugated complex) from GO in the Co_1_@GO (Fig. 1 and Supplementary Fig. 1). Furthermore, the peak position at 1.47 Å is in good agreement with that of Co_3_O_4_, suggesting that the Co atom sites in Co_1_@GO are at a high oxidation state^38^, from +2 to +3, which should show higher activity than that of a low oxidation state^39^. This result is in agreement with the XPS results, suggesting high electrochemical performance for Co_1_@GO (vide infra). The fitting results of the R space in Fig. 3c and the k-space in Supplementary Fig. 7 are consistent with the Co-O_3_-C structure in DFT calculation results (vide infra), and also indicate the existence of the Co-O-C electron conjugated complex in the Co_1_@GO.

### The electrosynthesis performance of H_2_O_2_

**Fig. 4:**
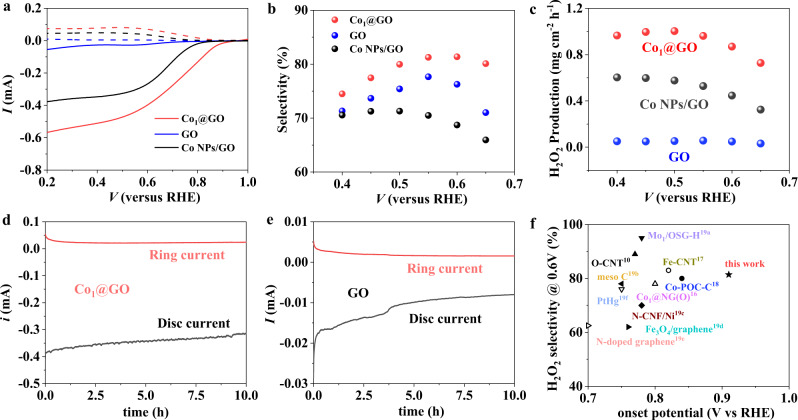
Oxygen reduction performance of Co_1_@GO and GO. **a**, **b** Oxygen reduction performance of Co_1_@GO and GO in 0.1 M KOH at 1600 rpm (solid lines) and H_2_O_2_ product currents (ring electrode, dashed lines). **c** Electrosynthesis of H_2_O_2_ on Co_1_@GO and GO in O_2_‐saturated 0.10 M KOH electrolyte. **d**, **e** Stability tests of Co_1_@GO and GO at 0.1 M KOH. **f** Comparison of the onset potential and selectivity for electrosynthesis H_2_O_2_ on Co_1_@GO and other reported catalysts at 0.6 V (RHE).

**Fig. 5:**
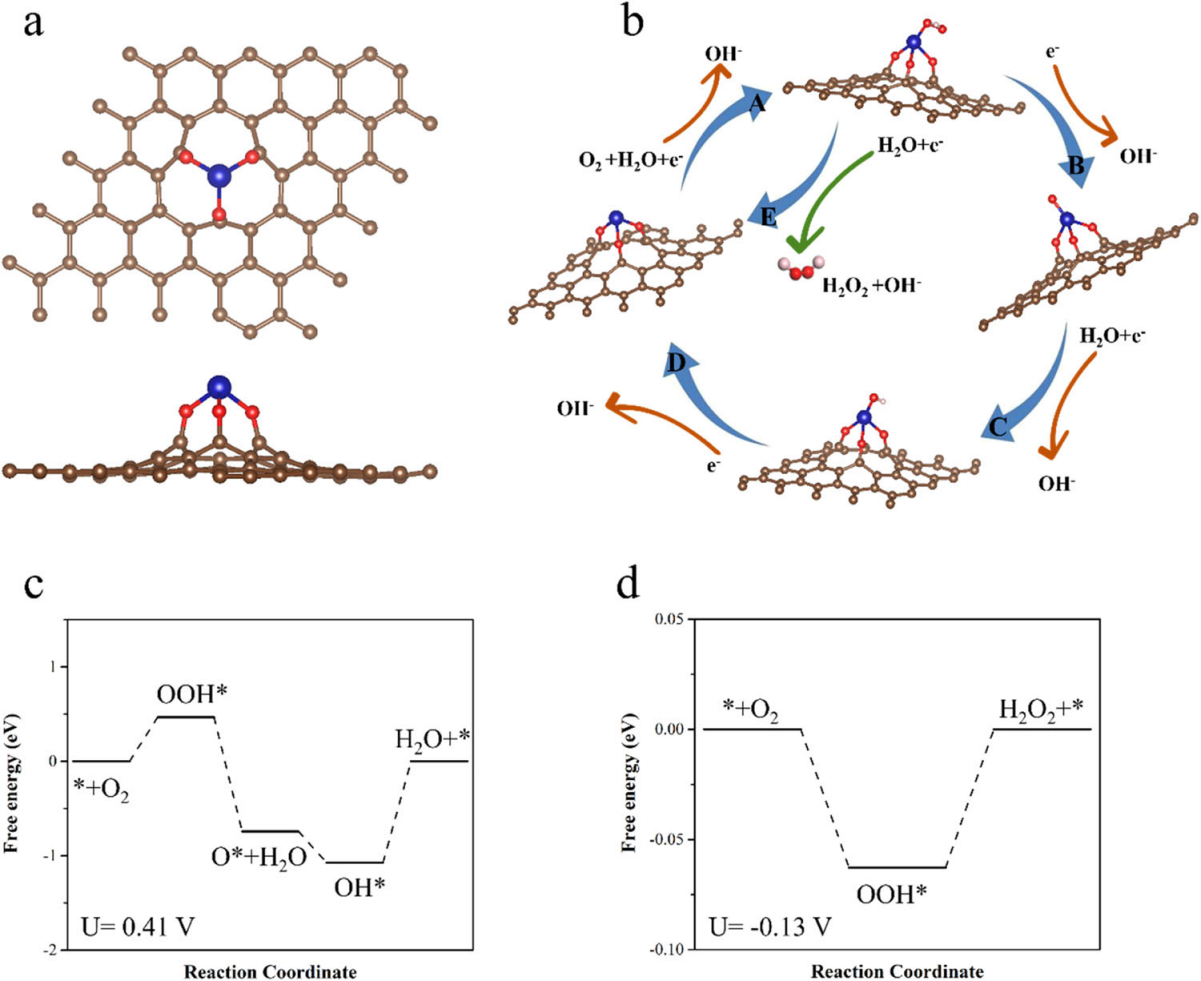
Mechanistic investigation through DFT. **a** The model of Co-O_3_-C structure used for DFT calculation. The brown, red and blue balls represent C, O and Co atoms, respectively. **b** The reaction cycle scheme of the intermediates in four-electron and two-electron ORR process on the Co-O_3_ catalytic center. **c** Free energy diagrams of four-electron under equilibrium state (*U* = 0.41 V), and **d** free energy diagrams of two-electron ORR under equilibrium state (*U* = −0.13 V).

To investigate the electrochemical performance of single Co atom sites and the possible influence of the oxygen functional group in Co_1_@GO, we performed the ORR measurements on Co_1_@GO, Co NPs/GO and GO in 0.1 M KOH (Fig. 4) using a rotating ring-disk electrode (RRDE). Figure 4a reproduces the linear sweep voltammetry (LSV) curves for Co_1_@GO, Co NPs/GO and GO in the 0.1 M KOH solution, in which the disk electrode detected the oxygen reduction currents while the Pt ring electrode monitored the yield of H_2_O_2_. As can be seen, Co_1_@GO possesses a higher ORR reactivity with an onset potential of 0.91 V versus a reversible hydrogen electrode (RHE) and a diffusion-limited current of ~0.5 mA, which are much higher than the corresponding values of 0.68 V and 0.028 mA for GO, 0.81 V and 0.34 mA for Co NPs/GO, and 0.76 V and 0.19 mA for Co clusters/GO (Supplementary Figs. 8 and 9). The high onset potential of Co_1_@GO is attributed to the synergistic effect between the O-bonded single Co atoms and adjacent oxygen functional groups (C-O bond) of the GO, which could decrease the free energy (−0.06 eV) of OOH* (Fig. 5d). The low free energy could improve the reactivity of ORR performance, thus the enhanced onset potential. The production of H_2_O_2_ was quantified at 1.2 V by the Pt ring electrode, which avoided the ORR current on the ring with H_2_O_2_ oxidation only. Besides, Fig. 4a shows a much higher ring current for Co_1_@GO than those of GO and Co NPs/GO, indicating that the presence of single Co atoms also enhanced the yield of H_2_O_2_. Figure 4b, c shows the selectivity and the yield of H_2_O_2_, respectively, for Co_1_@GO, GO and Co NPs/GO. As can be seen, the H_2_O_2_ selectivity of Co_1_@GO, GO and Co NPs/GO are 81.4%, 76.3%, and 68.7% at 0.6 V, respectively. The observed relatively small change in the selectivity for these samples indicates that the C-O may be the important factor for the selectivity. In addition, the Co_1_@GO shows a H_2_O_2_ yield of 1.0 mg cm^−2^ h^−1^ at 0.50 V, which is 19-fold enhancements compared with GO (0.052 mg cm^−2^ h^−1^) and about 2-fold improvement compared with Co NPs/GO (0.55 mg cm^−2^ h^−1^). The observed big change in the H_2_O_2_ yield among these samples suggests that the Co-O is the main reason for the activity for the 2e ORR reactivity. These results indicate that single Co atoms on the GO support could enhance the 2e ORR reactivity and improve the H_2_O_2_ selectivity, simultaneously.

Importantly, Co_1_@GO also maintains a high H_2_O_2_ selectivity and activity when applied to a two-electrode system. In 0.1 M KOH, Co_1_@GO delivers a steady-state H_2_O_2_ at 0.76 V with corresponding to an H_2_O_2_ production rate of ~5.7 mol g^−1^ h^−1^ or 28 mol m^−2^ h^−1^ (Supplementary Figs. 10 and 11). To our best knowledge, the onset potential and H_2_O_2_ production performance of Co_1_@GO are superior to most reported catalysts, such as Co_1_@NG(O) (0.42 mol g^−1^ h^−1^)^40^, Fe-CNT (1.6 mol g^−1^ h^−1^)^41^ and Co-POC-O (2.98 mol m^−2^ h^−1^)^42^ (Supplementary Table 1). Figure 4d, e presents the stability results, showing a disk and ring current of 0.32 and 0.024 mA, respectively, for Co_1_@GO at 36,000 s, which are 40 and 3.6 times of the corresponding date for GO (disk current of 0.008 mA and ring current of 0.0067 mA). Clearly, therefore, Co_1_@GO is a superb catalyst for the electrochemical generation of H_2_O_2_.

Figure 4f shows the onset potential versus the H_2_O_2_ selectivity for Co_1_@GO, along with the corresponding data from various previously reported catalysts for comparison.^31,40–50^ As can be seen, Co_1_@GO has the most positive potential with a relatively high H_2_O_2_ selectivity. For Co_1_@GO, the high oxidation state of single Co atoms and oxygen functional group of GO in the Co-O-C electron conjugated complex contributed to the high reactivity and high selectivity for electrosynthesis of H_2_O_2_. As shown in Supplementary Fig. 12, Co_1_@GO, Co NPs/GO, Co clusters/GO, and GO all show electron transfer numbers within the range of 2.3–2.7 over the potential from 0.4 to 0.7 V, indicating a 2e pathway for the oxygen reduction. The Co_1_@GO maintains the lowest electron transfer numbers, demonstrating again that synergistic effects between the O-bonded single Co atoms and adjacent oxygen functional groups (C-O bond) of the GO in the Co_1_@GO have significantly improved the kinetics for 2e ORR. Clearly, therefore, anchoring single Co atoms of a high oxidation state onto a GO support could afford a feasible strategy to develop advanced electrocatalysts with excellent reactivity, high selectivity, and good stability toward electrosynthesis of H_2_O_2_.

### Mechanistic investigation on H_2_O_2_ electrogeneration

To obtain a better understanding of the Co-O-C active centers and more insights into the catalytic mechanism of the Co_1_@GO catalysts, we further performed DFT calculations (Supplementary Methods) to determine the free energy, overpotential, and reaction pathway for ORR on Co_1_@GO. According to the experimental results for the coordination environment near the Co metal, the Co-O-C electron conjugated complex is detected as the active center in the Co_1_@GO catalyst. Therefore, we built DFT models with possible Co-O structures (Supplementary Fig. 13 and Fig. 5a). With the DFT calculations, the catalytic center is identified as Co-O_3_-C structure (Fig. 5a). The formation energy of this structure is −2.78 eV, indicating the thermodynamically spontaneous formation of the structures. The typical ORR cycle of four-electron pathway producing H_2_O (A-B-C-D) and two-electron pathway producing H_2_O_2_ (A-E) on the Co-O_3_ active center is illustrated in Fig. 4b. The free energy diagrams of four-electron and two-electron ORR for the Co-O_3_-C structure are shown in Fig. 5c, d, respectively. For four-electron ORR, the reaction center shows a quite low catalytic activity with overpotential of 1.07 V. The rate-limiting step of four-electron ORR is identified to be the final step (OH* desorption), which is caused by the strong absorption of OH*. For two-electron ORR, the free energy change is extremely close to that of the ideal catalyst, and the overpotential is only 0.06 V, showing a high activity of producing H_2_O_2_. Thus, from the results of DFT calculations, the two-electron ORR is favored for the identified catalytic structure, consistent with the experimental results of high reactivity and selectivity of Co_1_@GO for electroreduction of O_2_ to H_2_O_2_ via a 2e pathway.

The free energy diagrams for Co-O_4_-C structures (Supplementary Fig. 13) were also calculated and shown in Supplementary Fig. 14, and their formation energy and overpotential values considered are listed in Supplementary Tables 2 and 3, respectively. It is noted that the Co-O_4_-C-(OH)_4_ structure shown in Supplementary Fig. 13c also has good activity and selectivity of two-electron ORR toward H_2_O_2_, with four-electron overpotentials of 0.88 V and two-electron overpotential of 0.01 V, which is another possible active center. The density of states (DOS) and Bader charge distribution of the Co-O_3_-C structure and other Co-O_4_ structures are also analyzed and shown in Supplementary Figs. 15 and 16, respectively. In general, the metal ion Co in Co-O_4_ structures carries a higher charge than that on Co-O_3_ one, which may lead to stronger adsorption to the intermediates. The high electronic LDOS of the Co atom in Co-O_3_-C structure is close to the Fermi level, promoting the electron transfer from the active center to the absorbates during the reaction and leading to unique two-electron ORR activity, whereas there is a large bandgap in the Co-O_4_-C structures, which inhibits the electron transfer from the graphene sheet and consequently depresses the 2 electron ORR. Therefore, Co-O_3_-C structure is more possibly the active center for H_2_O_2_ production via a 2e pathway for ORR.

## Conclusion

In summary, we have demonstrated that high oxidation of single Co atoms anchored on a GO support (i.e., Co_1_@GO) exhibited excellent performance for electrosynthesis of H_2_O_2_ from ORR via a 2e pathway. The newly developed Co_1_@GO possessed a unique novel Co-O-C electron conjugated complex as the active center with synergistic effects between the O-bonded single Co atoms and adjacent oxygen functional groups (C-O bond) of the GO. Consequently, Co_1_@GO delivered the highest reactivity and selectivity when compared with GO and reported catalysts (cf. Fig. 3) with good stability. DFT calculations confirmed Co-O_3_-C as the catalytic center in Co_1_@GO. This work represents a breakthrough in the electrosynthesis of H_2_O_2_ by rationally designing and developing highly effective, selective, and stable electrocatalysts with new active centers. The methodology developed in this study offers a novel and efficient approach for developing new electrocatalysts for various electrochemical reactions.

## Methods

### Synthesis of Co_1_@GO samples

Co_1_@GO was prepared through the dispersion of 6.2 mg CoCl_2_ and 50 mg GO in Millipore water (18.2 MΩ·cm) under ultrasonication for 30 min (Ultrasonic baths, 480 W, Bandelin Electonic). The resultant mixture was freeze-dried (Alpha 1–2 LDplus Entry Freeze Dryer, Refrigeration system 0.43 kW, Martin Christ) for 48 h to remove water, followed by thermal treatment at 300 °C for 2 h in 5 vol% H_2_ in N_2_, forming Co nanoparticles (Co NPs/GO). The resultant powder was leached in 2 M HCl for 24 h, forming Co clusters (Co clusters/GO), and then 2 M HNO_3_ for 24 h to remove Co clusters. Finally, the sample was collected by centrifugation, washed with water and ethanol several times. The electrocatalyst thus produced was designated as Co_1_@GO. For comparison, GO was prepared through the same procedure without the addition of CoCl_2_.

### Structural characterization

XRD results were recorded by a powder XRD (GBC MMA diffractometer) with Cu Kα radiation at the scan rate of 2° min^−1^. XPS spectra were recorded on Thermo ESCALAB250i XPS by using Al Kα radiation, which was fixed in an analyzer transmission mode. The XAS results of Co K-edge were collected at an XAS station of the Beijing Synchrotron Radiation Facility. The morphological study of the newly developed catalysts was performed on a scanning TEM (JEOL ARM-200F, 200 keV).

### Electrochemical measurements

The electrochemical performance of the resultant catalysts was carried out by using a three-electrode system at an electrochemical workstation (CHI 760 E, CH Instrument, USA). A Pt wire was used as the counter electrode and a saturated calomel electrode was applied as the reference electrode. The working electrode was using a RRDE. The ring electrode was Pt ring with an outside diameter of 0.75 mm and an inside diameter of 6.5 mm; the disk electrode was a glass carbon disk with a diameter of 5.0 mm. The slurry was prepared by adding 5.0 mg catalysts into a mixture of 50 μL Nafion and 950 μL ethanol, and then was sonicated for 1 h to form a homogenous dispersion. 5 μL of the ink was dropped on the disk electrode. The working electrode was ready for testing after the solvent evaporated. The potential was calibrated to a RHE by applying the following equation:

$${{{{{\rm{E}}}}}}\,({{{{{\rm{RHE}}}}}})={{{{{\rm{E}}}}}}({{{{{\rm{SCE}}}}}})+0{{{{{\rm{.224V}}}}}}+0.0592\,{{{{{\rm{pH}}}}}}$$

All the tests were carried out in an O_2_ saturated 0.1 M KOH solution at room temperature with a rotated rate of 1600 rpm. LSV was performed at a scan speed of 10 mV/s, and the potential of the ring electrode was set at 1.5 V (vs. RHE) to detect the production of H_2_O_2_. The selectivity, productivity, and electron transfer number were calculated by the following two equations:

$${{{{{{\rm{H}}}}}}}_{2}{{{{{{\rm{O}}}}}}}_{2}\;{{{{{\rm{selectivity}}}}}}{:}{{{{{{\rm{H}}}}}}}_{2}{{{{{{\rm{O}}}}}}}_{2}( \% )=200\times ({i}_{r}/N)/({i}_{d}+{i}_{r}/N)$$

$${{{{{{\rm{H}}}}}}}_{2}{{{{{{\rm{O}}}}}}}_{2}\;{{{{{\rm{productivity}}}}}}\,({{{{{\rm{mg}}}}}}\,{{{{{{\rm{cm}}}}}}}^{-2}{{{{{{\rm{h}}}}}}}^{-1})=1/2\times ({i}_{r}/N)\times {M}_{{{{{{{\rm{H}}}}}}}_{2}{{{{{{\rm{O}}}}}}}_{2}}\times 3600/({{{{{\rm{F}}}}}}/A)$$

$${{{{{\rm{Electron}}}}}}\,{{{{{\rm{transfer}}}}}}\,{{{{{\rm{number}}}}}}\,(n)=\frac{4\,{i}_{r}}{{i}_{r}+{i}_{d}/N}$$

*i*_*r*_ was the ring current and *i*_*d*_ was the disk current, *N* is the current collection efficiency of Pt ring electrode (*N* = 0.26), *M*_H2O2_ is the molecular weight of H_2_O_2_ (*M*_H2O2_ = 34.01 g mol^−1^), F is Faraday constant (F = 96485.3 C mol^−1^), *A* is the area of disk electrode (*A* = 0.196 cm^−2^). The collection efficiency of the electrode defined as the fraction of product from the disk to the ring is 0.26.

The stability test of these catalysts was performed by the chronoamperometric at a constant potential of −0.77 V (vs. RHE).

The bulk H_2_O_2_ production in 0.1 M KOH of Co_1_@GO was conducted in an H-cell electrolyzer, with 0.5 mg cm^−2^ Co_1_@GO onto a 2 × 2 cm^2^ Freudenberg GDL electrode (Fuel Cell Store) as the cathode, and Pt foil as the anode for H_2_O electro-oxidation. A Fumasep FAA-3-PK-130 anion exchange membrane (Fuel Cell Store) was used to separate the chambers. The H_2_O_2_ concentration can be quantified by Ce(SO_4_)_2_ titration based colorimetric method (2Ce^4+^ + H_2_O_2_ → 2Ce^3+^ + 2 H^+^ + O_2_)^31^. The H_2_O_2_ concentration-absorbance liner was calibrated by adding certain amount of commercial H_2_O_2_ solution with 0.5 mM Ce(SO_4_)_2_. The Ce^4+^/H_2_O_2_ concentration was measured on a Cary 5000 UV-Vis-NIR spectrometer (Agilent) and the absorption at 320 nm wavelength was applied to determine its concentration.

### Computational method

The DFT simulation on the free energies and overpotentials of two-electron ORR and four-electron ORR was carried out by DFT using the Vienna Ab initio simulation package (VASP)^51,52^, where the soft projector-augmented wave and Perdew–Burke–Ernzerhof exchange correlation were implemented^53^. The unit cells of the structures used in this study are composed of a single-layer graphene sheet embedded with Co and O atoms with the unit size set to 12.82 Å × 12.34 Å × 20.06 Å. Spin-polarization was considered in all calculations. The cutoff energy was set to 550 eV and the geometries were fully relaxed until the residual force convergence value on each atom being less than 0.01 eV Å^−1^. The structures were sampled by a 3 × 3 × 1 k-point Monkhorst-Pack k-point mesh. In the calculation of reaction substrate and absorbates, the implicit solvent effect is considered to simulate the aqueous environment by using the VASPsol code. Since Co is 3d-transition metal, the Hubbard U (DFT+U) corrections were also considered in the calculations^54^.

## Supplementary information

Supplementary Material

## Data Availability

The data that support the findings of this study are available from the corresponding author upon reasonable request.
