# Supplementary material for: Highly efficient and selective electrocatalytic hydrogen peroxide production on Co-O-C active centers on graphene oxide
Source: Commun Chem. 2022 Mar 28;5:43. doi: 10.1038/s42004-022-00645-z (PMC9814078; doi:10.1038/s42004-022-00645-z)
Supplement: Supplementary file 1 — Supplementary Material [file 42004_2022_645_MOESM1_ESM.pdf]

Supplementary Information

Highly efficient and selective electrocatalytic hydrogen peroxide production on Co-O-C active centers on graphene oxide

Bin-Wei Zhang,[1] Tao Zheng,[2] Yun-Xiao Wang,[3] Yi Du,[3] Sheng-Qi Chu,[4] Zhenhai Xia,[2] Rose Amal,[1] Shi-Xue Dou,[3] and Liming Dai[1,*]

[1] Australian Carbon Materials Centre (A-CMC), School of Chemical Engineering, The University of New South Wales Sydney, NSW 2052, Australia. E-mail: l.dai@unsw.edu.au

[2] Department of Materials Science and Engineering, Department of Chemistry, University of North Texas, Denton, TX 76203, USA

[3] Institute for Superconducting and Electronic Materials, Australian Institute of Innovative Materials, University of Wollongong, Innovation Campus, Squires Way, North Wollongong, New South Wales 2500, Australia.

[4] Beijing Synchrotron Radiation Facility, Institute of High Energy Physics, Chinese Academy of Sciences, Beijing 100049, People's Republic of China.

Supplementary Methods

1. Calculation methods

1.1 Oxygen evolution reaction

In an alkaline environment, the overall 4-electron oxygen evolution reaction (ORR) with final product H_2O can be written as:

$$O_2 + 2H_2O(l) + 4e^- \rightarrow 4OH^- \quad \text{(S1)}$$

The associative mechanism of 4-electron ORR goes through the following elementary steps:

$$O_2 + * + H_2O(l) + e^- \rightarrow OOH^* + OH^- \quad \text{(S2)}$$

$$OOH^* + e^- \rightarrow O^* + OH^- \quad \text{(S3)}$$

$$O^* + H_2O(l) + e^- \rightarrow OH^* + OH^- \quad \text{(S4)}$$

$$OH^* + e^- \rightarrow OH^- + * \quad \text{(S5)}$$

As for 2- electron ORR with final product H_2O_2, the overall reaction can be written as:

$$O_2 + 2H_2O(l) + 4e^- \rightarrow H_2O_2(l) + 2OH^- \quad \text{(S6)}$$

And the associative mechanism of 2-electron ORR goes through the following elementary steps:

$$O_2 + * + H_2O(l) + e^- \rightarrow OOH^* + OH^- \quad \text{(S7)}$$

$$OOH^* + H_2O(l) + e^- \rightarrow H_2O_2(l) + OH^- + * \quad \text{(S8)}$$

The free energy change of each step can be obtained by[1]:

$$\Delta G = \Delta E^{DFT} + \Delta ZPE - T\Delta S + \Delta G_U + \Delta G_{pH} \quad \text{(S9)}$$

where ΔE^{DFT} is the DFT-determined energy change of each step, T=293.15 K is the room temperature, and ΔS is the entropy change. ZPE is the zero-point energy of OH*, O* and OOH*, which is calculated with the contribution in the free energy expression considering only vibrational entropy frequencies. $\Delta G_U = -eU$ is the potential correction, where U is the potential at the electrode. ΔG_{pH} is the correction of the H^+ free energy by the concentration dependence of the entropy:

$$\Delta G_{pH} = -k_B T \ln[H^+] \quad \text{(S10)}$$

where k_b is the Boltzmann constant. The ORR overpotentials η in alkaline medium (pH=14), were determined by the following equations[2]:

$$\eta^{\text{4-electron ORR}} = max\{\Delta G_1, \Delta G_2, \Delta G_3, \Delta G_4\} \quad \text{(S11)}$$

$$\eta^{\text{2-electron ORR}} = max\{\Delta G_1, \Delta G_2\} \quad \text{(S12)}$$

where $\Delta G_1, \Delta G_2, \Delta G_3, \Delta G_4$ are the free energy change of each elementary step for 4-electron ORR under U = 0.41 V and 2-electron ORR under U = -0.13 V.

1.2 Formation energy

The formation energy of the proposed structures is estimated by[3]:

$$\Delta E = E_s + lE_C - \left(E_{Co} + E_g + mE_H + nE_O\right) \quad \text{(S13)}$$

where E_g and E_s are the energy of pure graphene and the catalytic structures calculated by DFT, respectively. E_O and E_H are the half chemical potential of O_2 and H_2 molecule, respectively. *n* and *m* are the number of O and H atoms in the catalytic structures. *l* is the number difference of C atom between pure graphene and the catalytic structure. E_{Co} is the free energy of isolated Co atom, and E_C is the average energy to each atom of pure graphene.

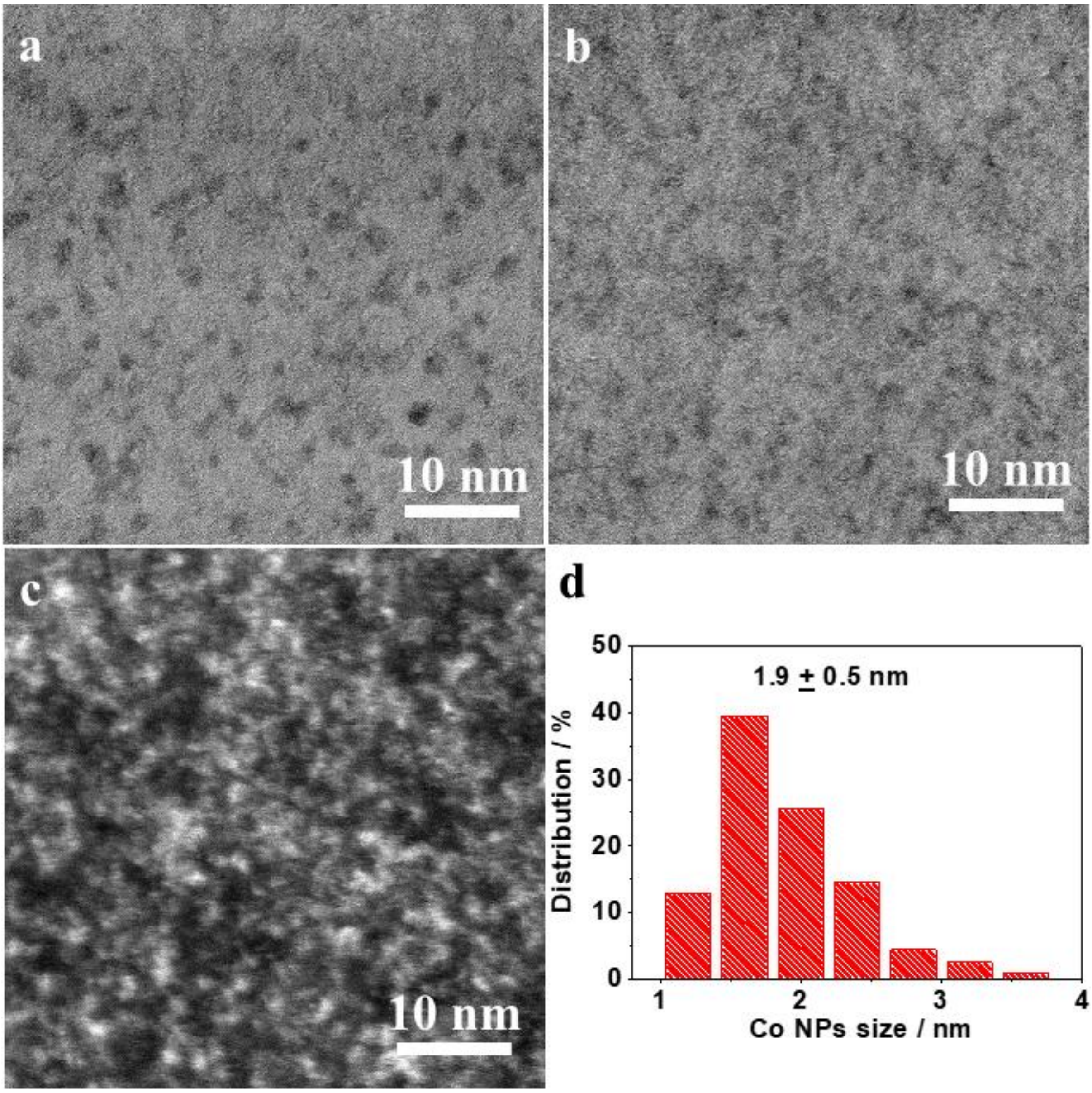

Supplementary Figure 1. a-c, Transmission electron microscope (TEM) and high-angle annular dark field – scanning transmission electron microscope (HAADF-STEM) images of Co nanoparticles on GO (Co NPs/GO). **d**, Histrogram showing Co NPs distribution based on a count of 200 Co NPs in the sample areas.

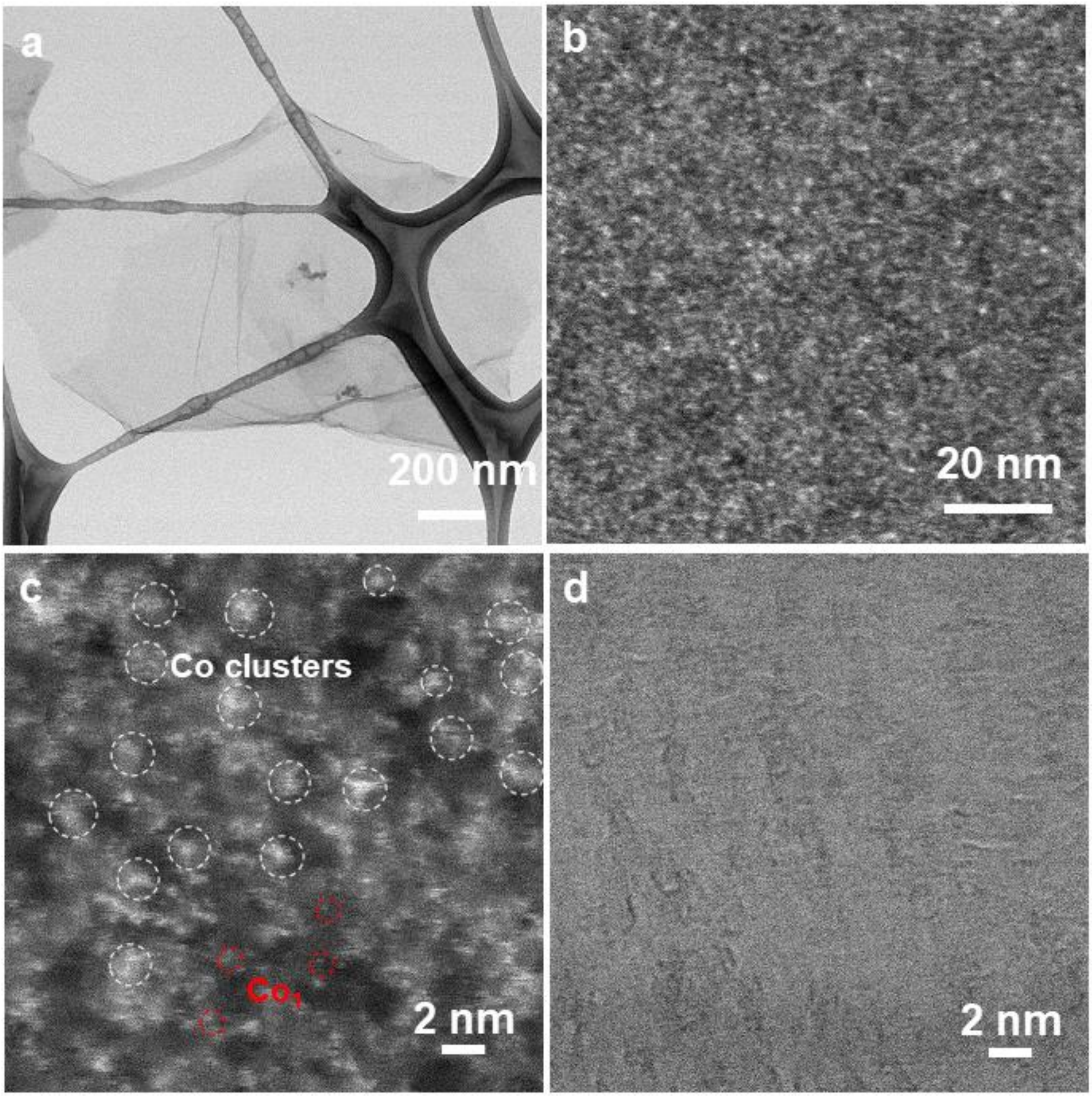

Supplementary Figure 2. a-d, Transmission electron microscope (TEM) and high-angle annular dark field – scanning transmission electron microscope (HAADF-STEM) images of Co clusters on GO (Co clusters/GO).

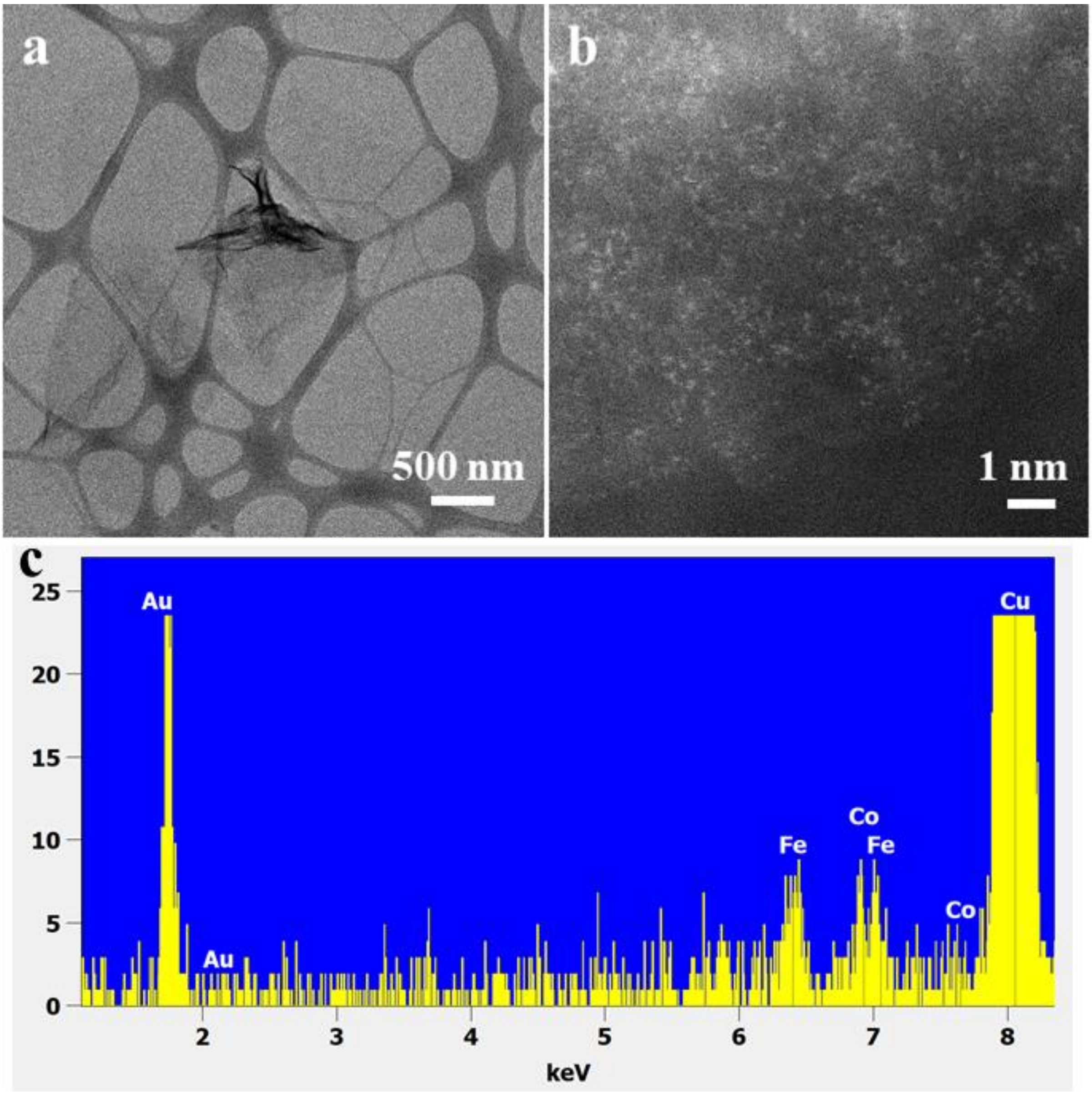

Supplementary Figure 3. a-b, TEM and HAADF-STEM images, and **c**, energy dispersive X-ray spectroscopy (EDS) result of Co_1@GO. Note that there may be some co-existing Fe signals as the EDS technology is usually difficult to distinguish the Fe and Co signals. The Fe impurity, if any, is intrinsically associated with the GO sample while the Au and Cu signals are arising from the Cu TEM grid and Au coating used during the sample preparation for TEM and HAADF-STEM imaging.

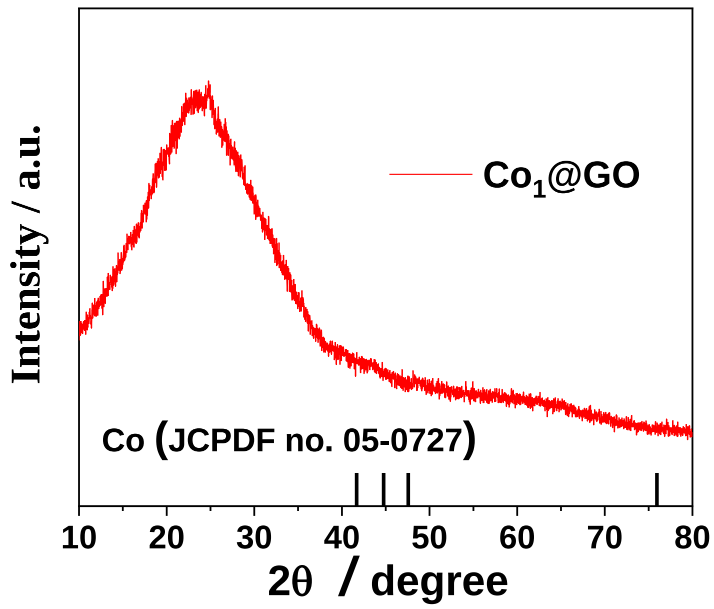

Supplementary Figure 4. XRD patterns of the Co_1@GO.

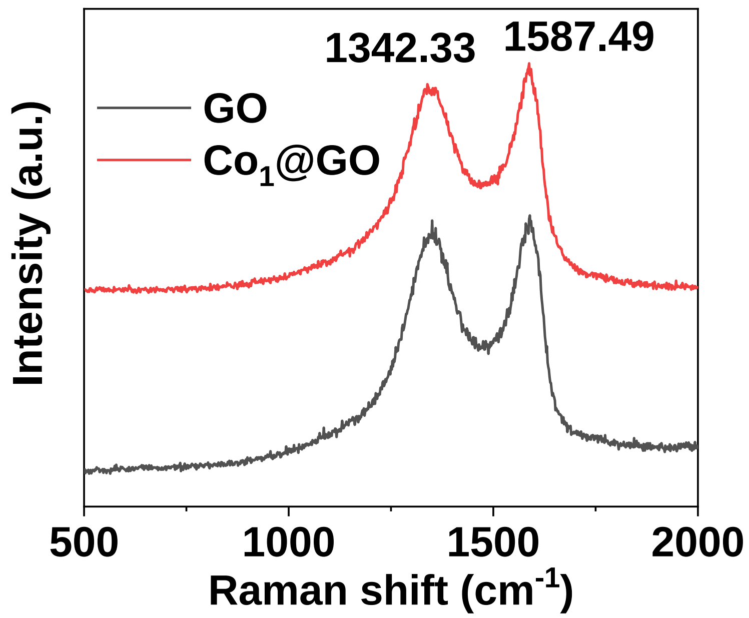

Supplementary Figure 5. Raman spectra of GO and Co_1@GO.

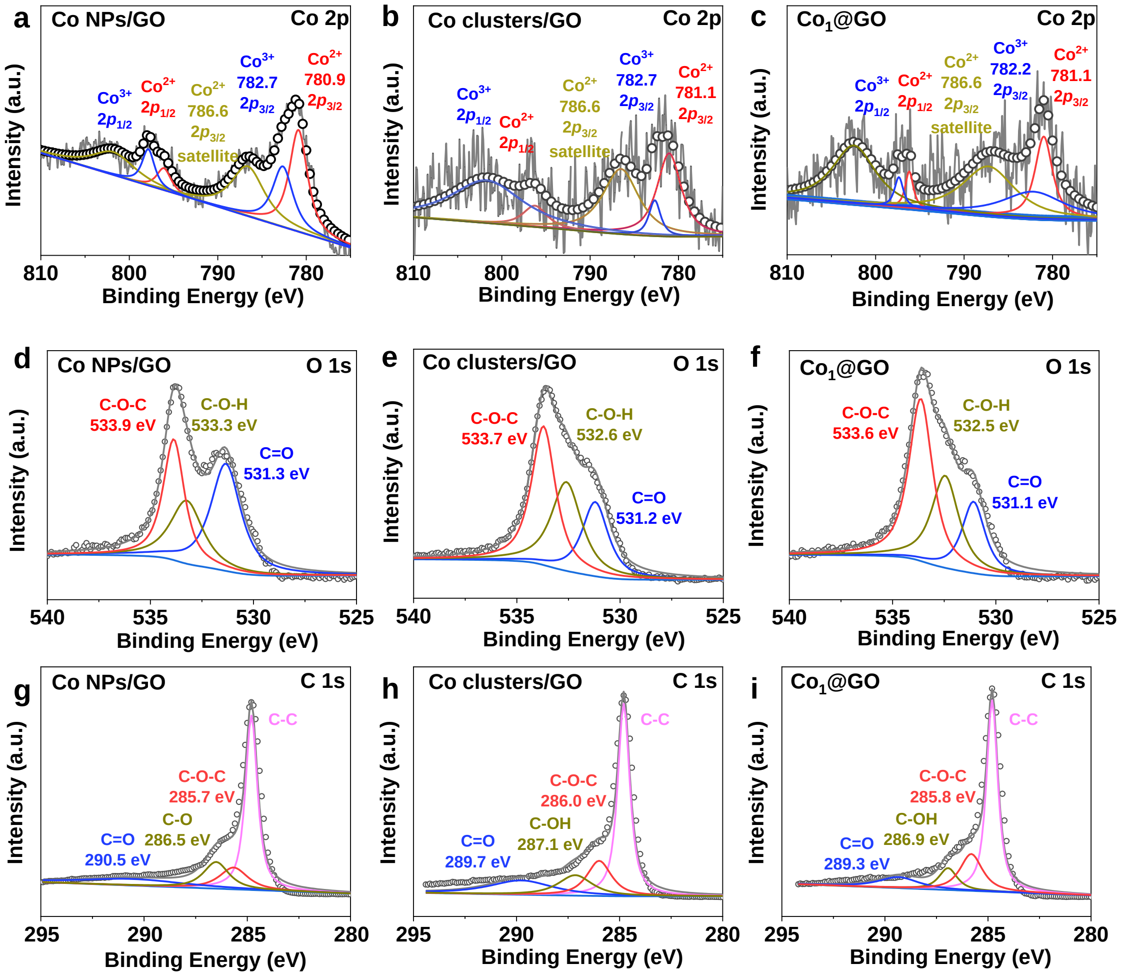

Supplementary Figure 6. (a)-(c) Co 2p XPS sepctra of Co NPs/GO, Co clusters/GO, and Co_1@GO. (d)-(f) O 1s XPS sepctra of Co NPs/GO, Co clusters/GO, and Co_1@GO. (g)-(i) C 1s XPS sepctra of Co NPs/GO, Co clusters/GO, and Co_1@GO.

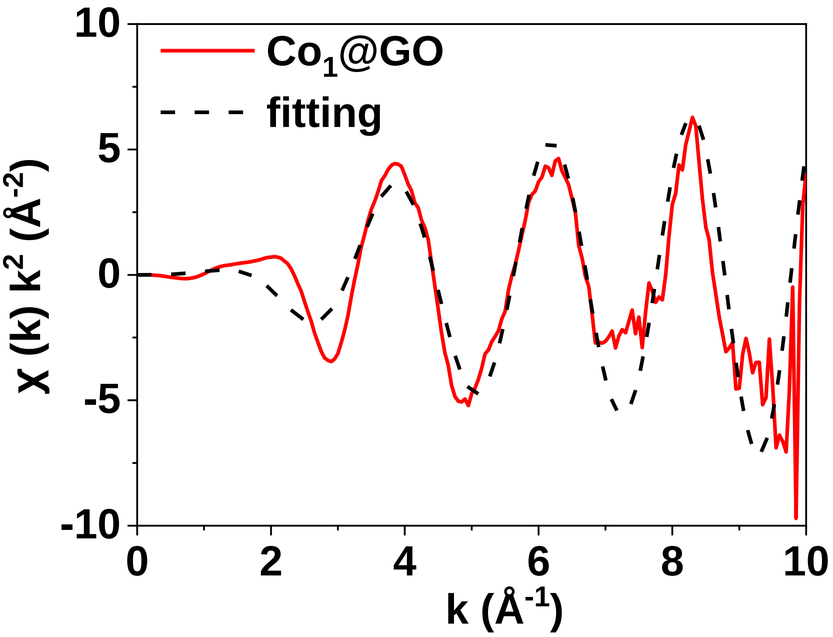

Supplementary Figure 7. Co K-edge k-space experimental EXAFS spectrum and fitted curves to Co_1@GO.

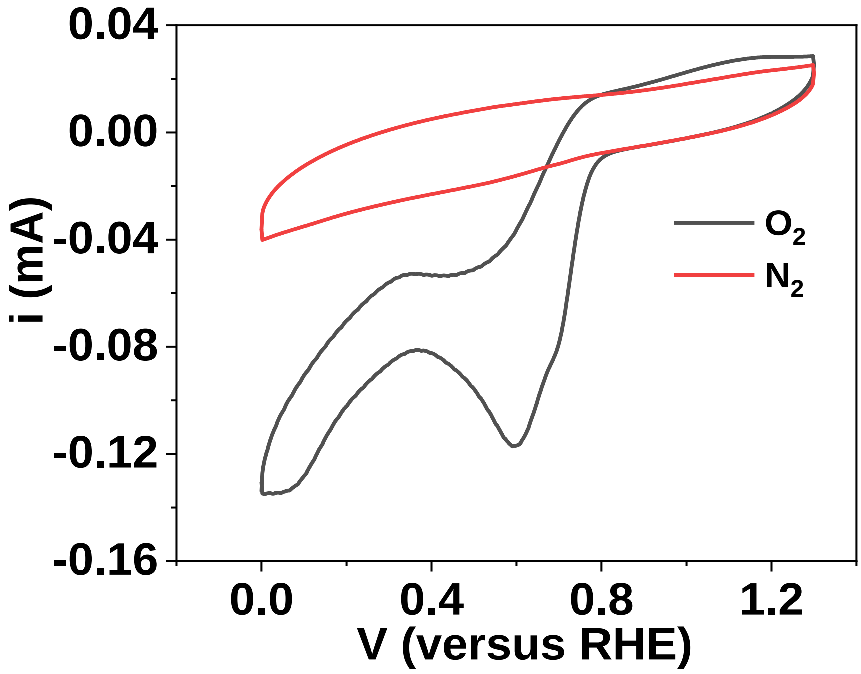

Supplementary Figure 8. Cyclic voltammetry of Co_1@GO sample in N_2 and O_2 saturated 0.1 M KOH at the scan speed of 50 mV s^{-1}.

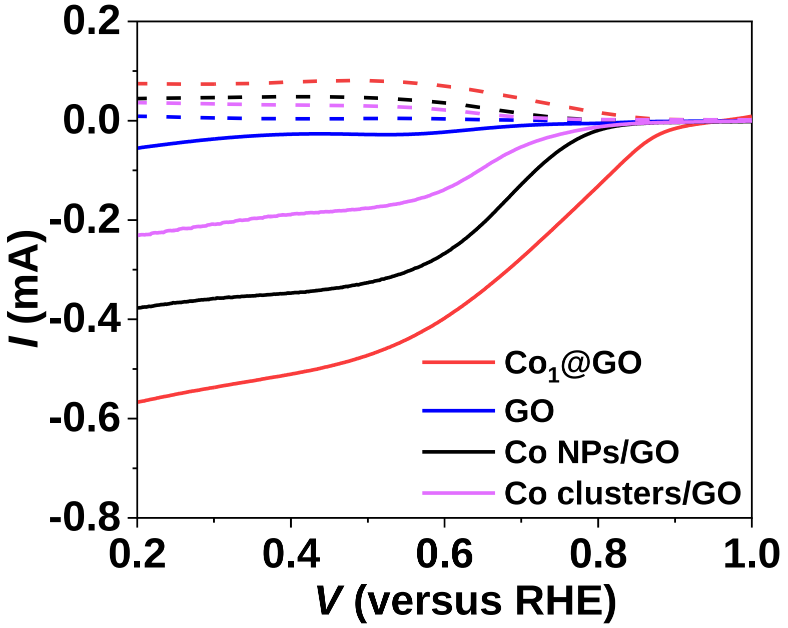

Supplementary Figure 9. Oxygen reduction performance of Co₁@GO, GO, Co NPs/GO, and Co clusters/GO in 0.1 M KOH at 1,600 r.p.m. (solid lines) and H_2O_2 product currents (ring electrode, dashed lines).

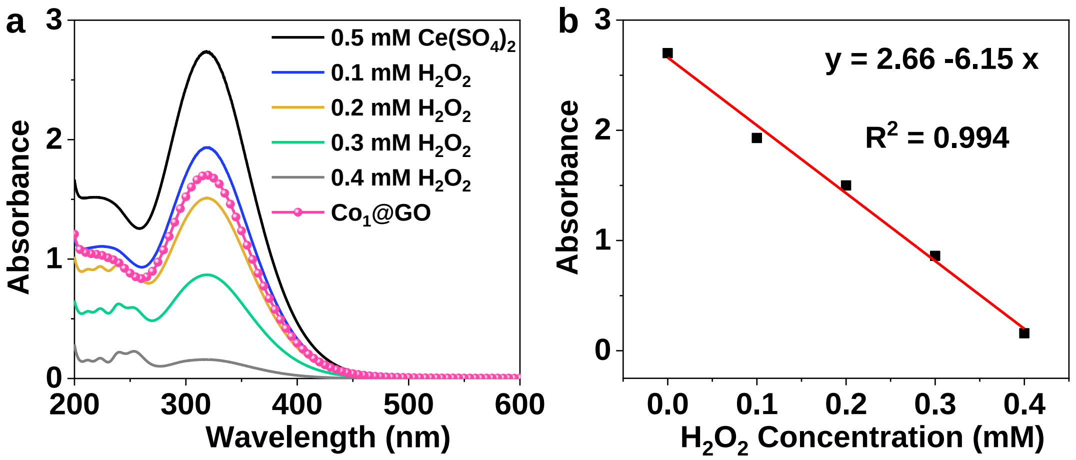

Supplementary Figure 10. (a) UV-Vis absorption spectra for cerium titration by known H_2O_2 concentration and Co_1@GO. (b) linear fitting of absorbance at 320 nm wavelength.

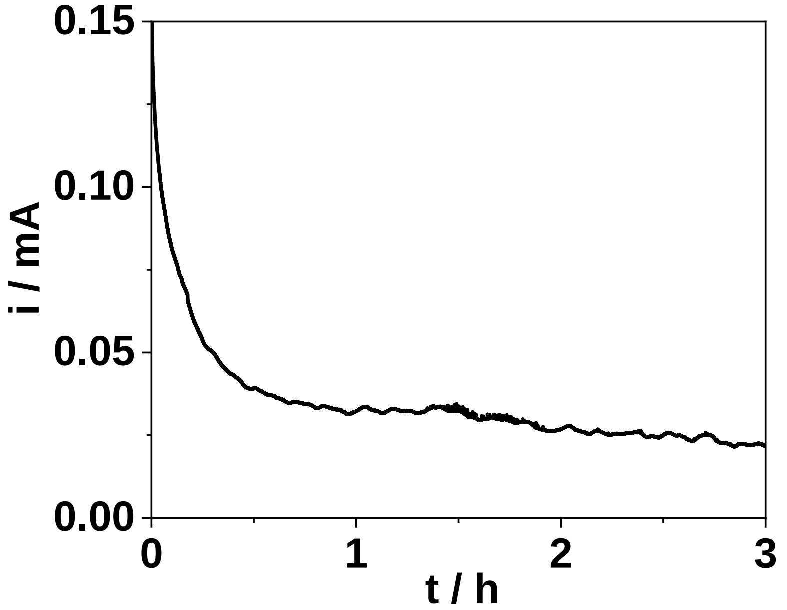

Supplementary Figure 11. Bulk electrolysis for H_2O_2 generation in a homemade H-cell electrolyzer, with 0.5 mg cm^{-2} Co_1@GO air-brushed onto a 2×2 cm^2 Freudenberg GDL electrode (Fuel Cell Store) as ORR cathode, with the Pt foil for water oxidation.

Supplementary Table 1. Electrochemical performance comparison of the Co_1@GO and other reported electrocatalysts for H_2O_2 electrochemical synthesis.

Sample	Two-electrode system for H_2O_2 productivity	Onset potential (RHE)	Selectivity	Electrolyte	Reference
**Co_1@GO**	5.7 mol g^{-1} h^{-1} (28 mol m^{-2} h^{-1}) @0.76 V	0.91 V	81.4% @0.6 V	0.1 M KOH	**This work**
**Co_1@NG(O)**	0.42 mol g^{-1} h^{-1} @0.6 V	0.8 V	72% @0.6 V	0.1 M KOH	[4]
**Fe-CNT**	1.6 mol g^{-1} h^{-1} (28 mol m^{-2} h^{-1}) @0.76V	0.82 V	94% @0.6 V	0.1 M KOH	[5]
**Co-POC-O**	2.98 mg cm^{-2} h^{-1}@0.70V	0.84 V	81% @0.6 V	0.1 M KOH	[6]
**Mo_1/OSG-H**	/	0.78 V	95% @0.6 V	0.1 M KOH	[7]
**O-CNT**	/	0.77 V	90% 0.6V	0.1 M KOH	[8]
**Meso C**	/	0.75 V	78% @0.6 V	0.1 M KOH	[9]
**N-CNF/Ni**	/	0.78 V	70% @0.6 V	0.5 M KOH	[10]
**Fe_3O_4/ graphene**	/	0.702 V	70% @0.6 V	1 M KOH	[11]
**N-doped graphene**	/	0.77 V	50% @0.6 V	0.1 M H_2SO_4	[12]
**PtHg**	/	0.75 V	95% @0.4 V	0.1 M $HClO_4$	[13]

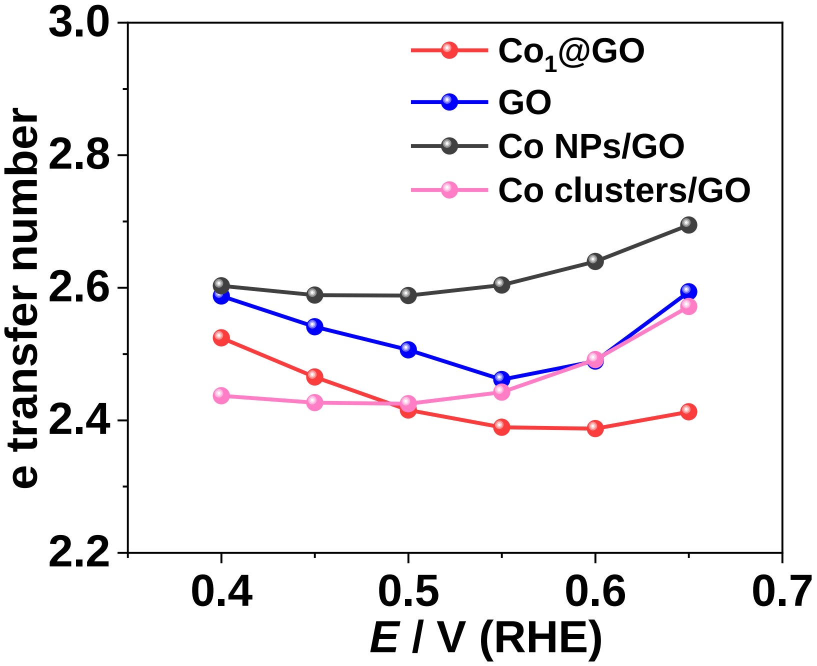

Supplementary Figure 12. Calculated H_2O_2 electron transfer number during potential sweep.

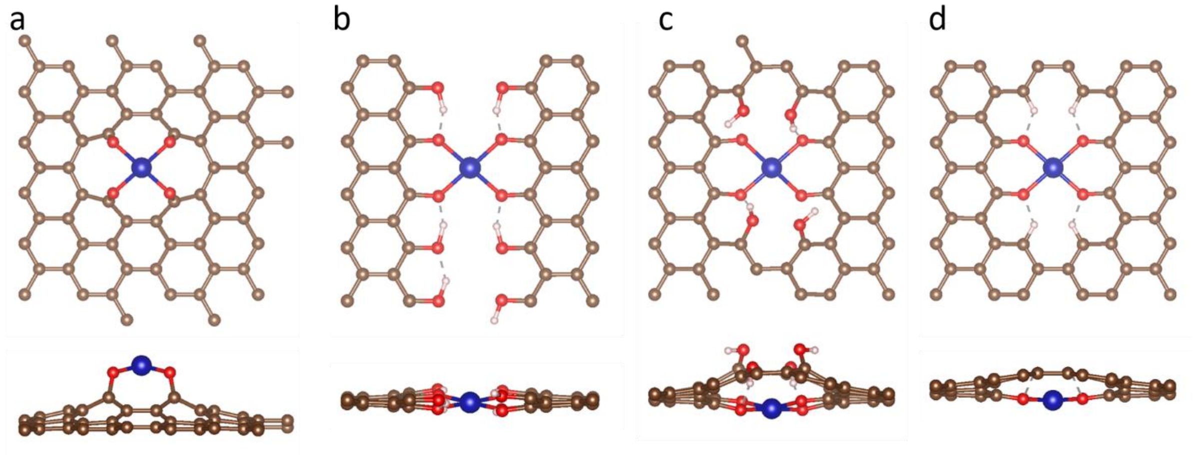

Supplementary Figure 13. Co-doped structures considered in DFT calculation. (a) Co-O_4-C structure. (b) Co-O_4-C-$(OH)_6$ structure. (c) Co-O_4-C-$(OH)_4$ structure. (d) Co-O_4-C-H_4 structure.

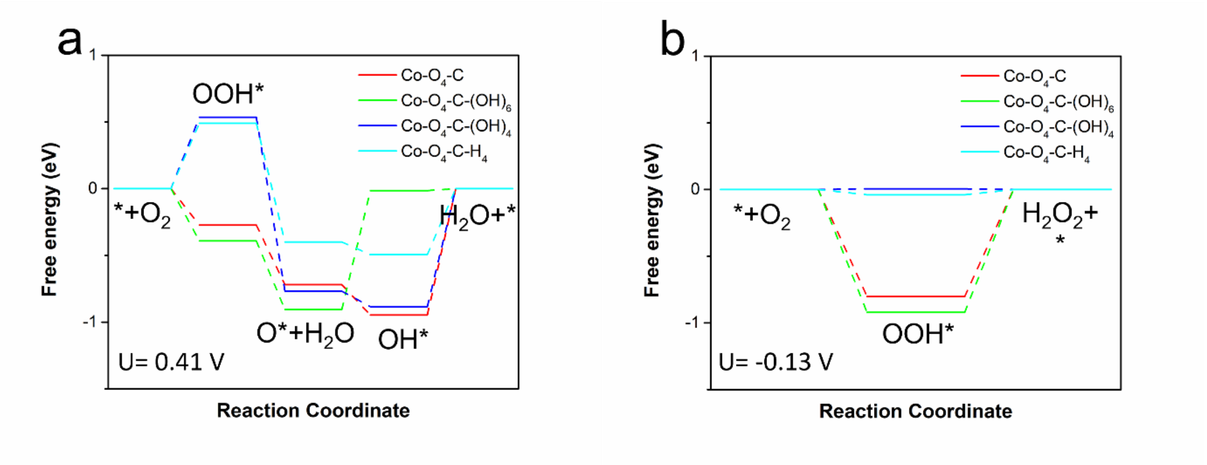

Supplementary Figure 14. Free energy diagrams of the Co-O-doped structures shown in Figure S12. (a) 4-electron ORR under equilibrium state (U=0.41 V). (b) 2-electron ORR under equilibrium state (U=-0.13 V).

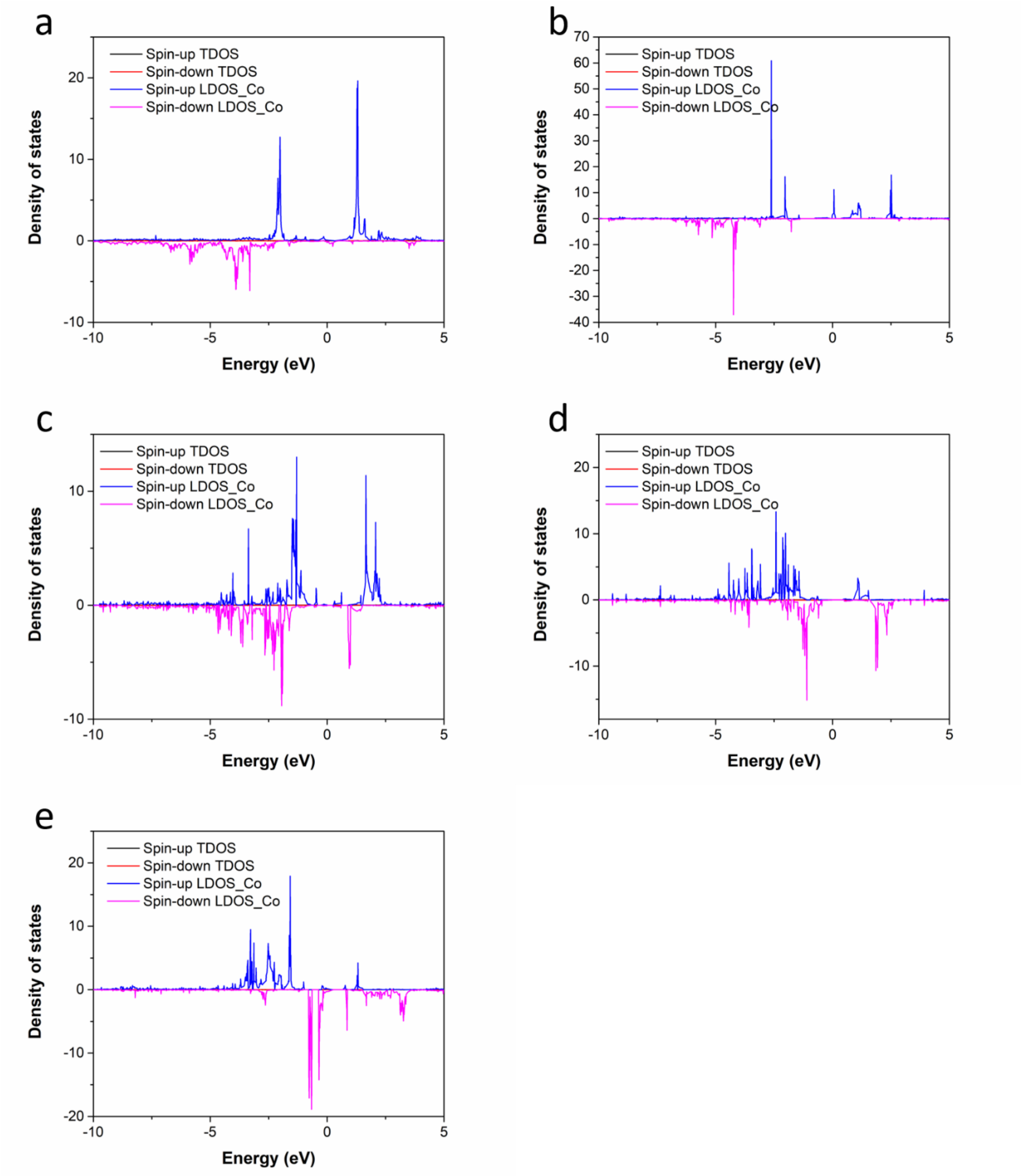

Supplementary Figure 15. Density of states (DOS) of the 4 structures listed in Figure S1 and the structure in Figure 1a. (a) Co-O_4-C structure. (b) Co-O_4-C-$(OH)_6$ structure. (c) Co-O_4-C-$(OH)_4$ structure. (d) Co-O_4-C-H_4 structure. (e) Co-O_3-C structure.

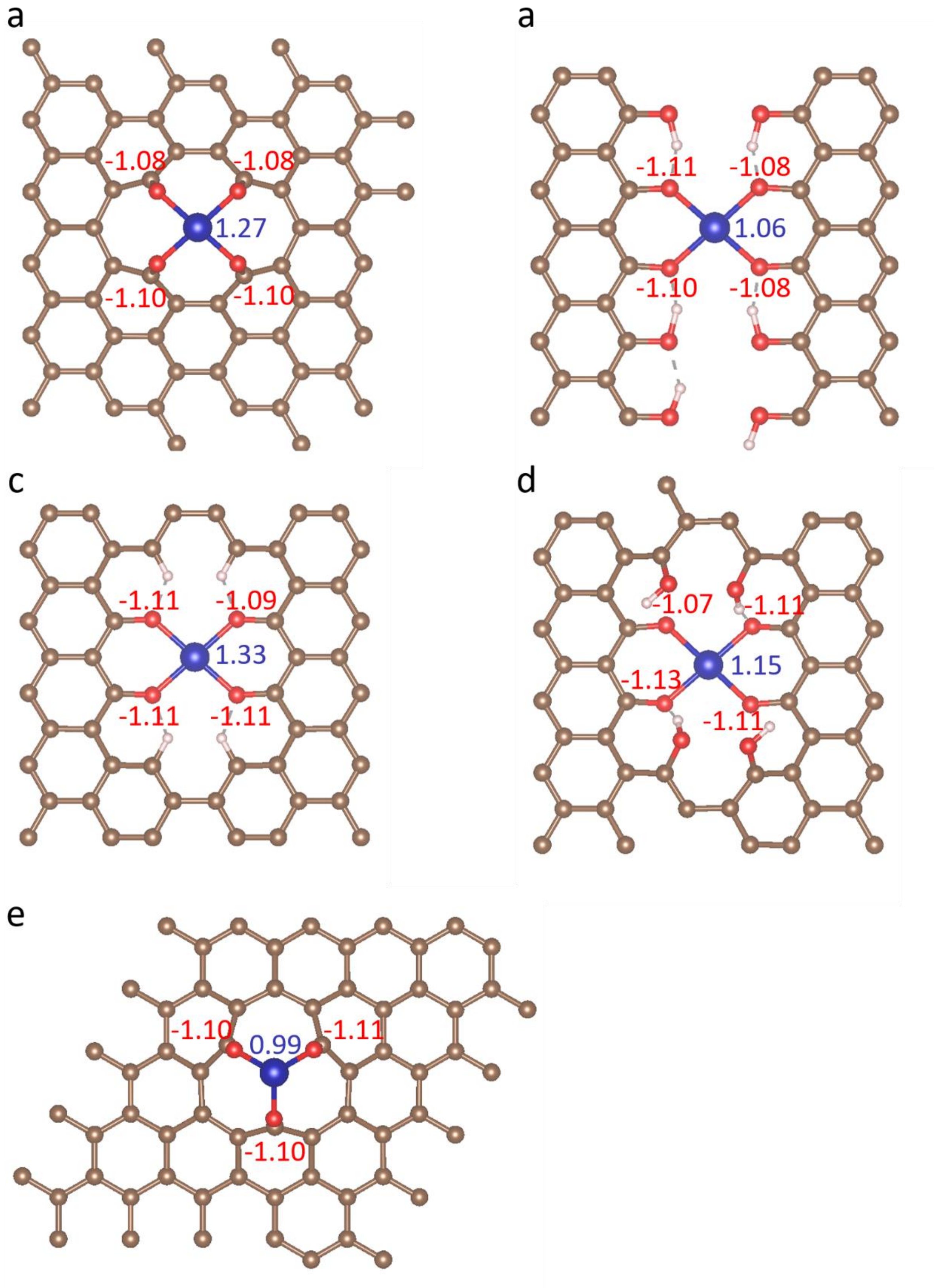

Supplementary Figure 16. Bader charge distribution the 4 structures listed in Figure S1 and the structure in Figure 1a. (a) Co-O_4-C structure. (b) Co-O_4-C-$(OH)_6$ structure. (c) Co-O_4-C-$(OH)_4$ structure. (d) Co-O_4-C-H_4 structure. (e) Co-O_3-C structure.

Supplementary Table 2. The formation energy values of the structures shown in Figure S1 calculated by Eq. (S12)

Structure	Co-O_4-C	Co-O_4-C-$(OH)_6$	Co-O_4-C-$(OH)_4$	Co-O_4-C-H_4
Formation energy (eV)	-3.90	-21.13	-12.69	-7.29

Supplementary Table 3. The values of 4e and 2e overpotential in the structures shown in Figure S1 and Figure 1a

Structure	Co-O_3-C	Co-O_4-C	Co-O_4-C-$(OH)_6$	Co-O_4-C-$(OH)_4$	Co-O_4-C-H_4
Overpotential of 4e ORR (V)	1.07	0.95	0.89	0.88	0.49
Overpotential of 2e ORR (V)	0.06	0.56	0.92	0.01	0.04

Supplementary References

1. Li, M., Zhang, L., Xu, Q., Niu, J. & Xia, Z. *J. Cata.* **314**, 66-72 (2014).
2. Man, I. C. *et al*. *ChemCatChem* **3**, 1159-1165 (2011).
3. Qi, W., Niu, J., Huang, Z., Chen, Z., Chen, X., Chen, F. & Tuo, J. *Int. J. Hydrogen Energy* **45**, 521-530 (2020).
4. Jung, E. et al. *Nat. Mater.* **19**, 436-442 (2020).
5. Jiang, K. *et al*. *Nat. Commun.* **10**, 3997 (2019).
6. Li, B.-Q., Zhao, C.-X., Liu, J.-N. & Zhang, Q. *Adv. Mater.* **31**, 1808173 (2019).
7. Tang, C., Jiao, Y., Shi, B., Liu, J.-N., Xie, Z., Chen, X., Zhang, Q. & Qiao, S.-Z. *Angew. Chem. Int. Ed.* **59**, 9171-9176 (2020).
8. Lu, Z. *et al*. *Nat. Catal.* **1**, 156-162 (2018).
9. Chen, S. *et al*. *ACS Sus. Chem. Eng.* **6**, 311-317 (2018).
10. Buan, M. E. M., Muthuswamy, N., Walmsley, J. C., Chen, D. & Rønning, M. *Carbon* **101**, 191-202 (2016).
11. Barros, W. R. P., Wei, Q., Zhang, G., Sun, S., Lanza, M. R. V. & Tavares, A. C. *Electrochim. Acta* **162**, 263-270 (2015).
12. Chen, C.-Y., Tang, C., Wang, H.-F., Chen, C.-M., Zhang, X., Huang, X. & Zhang, Q. *ChemSusChem* **9**, 1194-1199 (2016).
13. Siahrostami, S. *et al*. *Nat. Mater.* **12**, 1137 (2013).
